# Low-affinity CAR T cells exhibit reduced trogocytosis, preventing rapid antigen loss, and increasing CAR T cell expansion

**DOI:** 10.1038/s41375-022-01585-2

**Published:** 2022-04-30

**Authors:** Michael L. Olson, Erica R. Vander Mause, Sabarinath V. Radhakrishnan, Joshua D. Brody, Aaron P. Rapoport, Alana L. Welm, Djordje Atanackovic, Tim Luetkens

**Affiliations:** 1 Department of Pathology, University of Utah, Salt Lake City, UT USA; 2 Hematology and Hematologic Malignancies, Huntsman Cancer Institute, University of Utah, Salt Lake City, UT USA; 3 Department of Microbiology and Immunology, University of Maryland School of Medicine, Baltimore, MD USA; 4 Department of Medicine and Transplant/Cell Therapy Program, University of Maryland School of Medicine and Marlene and Stewart Greenebaum Comprehensive Cancer Center, Baltimore, MD USA; 5 Department of Pharmaceutics & Pharmaceutical Chemistry, University of Utah, Salt Lake City, UT USA; 6 Division of Hematology and Oncology, Medical College of Wisconsin, Milwaukee, WI USA; 7 Tisch Cancer Institute, Icahn School of Medicine at Mount Sinai, New York, NY USA; 8 Department of Oncological Sciences, Huntsman Cancer Institute, University of Utah, Salt Lake City, UT USA

**Keywords:** Preclinical research, Biotechnology

To the Editor:

Chimeric antigen receptor (CAR) T cells, specifically CAR T cells targeting CD19, have greatly improved outcomes for patients with B cell lymphoma. However, disease relapse remains a common occurrence in many patients [1, 2]. Different mechanisms of relapse have been demonstrated, such as CD19 open-reading frame mutations, upregulation of coinhibitory ligands, and CAR T cell exhaustion [3]. However, in many relapsed patients no clear immune escape mechanisms can be identified and additional processes, such as trogocytosis, may play an important role. Trogocytosis is a process originally described in the TCR context whereby TCR internalization upon binding of peptide/MHC results in stripping of MHC from antigen presenting cells and subsequent display of MHC on the surface of the recipient T cell [4]. Trogocytosis has since been observed in many facets of natural immunity and plays an important role in immune cell development and function [5]. Recently, CAR T cells were also shown to exhibit trogocytosis when co-cultured with different types of cancer cells, including CAR T cells based on the high-affinity CD19 binding domain FMC63, which were shown to strip CD19 from lymphoma cells and incorporate it into their own plasma membrane [6]. In addition, it was shown that T cells engineered to express CD19 can be killed by CD19 CAR T cells suggesting that such fratricide might also occur spontaneously after CAR T cells acquire CD19 by trogocytosis [6]. To date, no strategies exist to limit trogocytosis in the CAR T cell context to prevent fratricide and the emergence of antigen-negative tumor cells. We, therefore, aimed to develop an approach to limit CAR T cell-mediated trogocytosis. Trogocytosis has previously been shown to correlate with TCR-peptide/MHC avidity [7], and we hypothesized that CAR affinity may have a similar relationship to CAR T cell-mediated trogocytosis. Furthermore, as current iterations of antigen-binding domains used for the construction of CARs likely exceed the affinity required for efficient target cell killing [8], we hypothesized that it may be possible to separate the pro-tumorigenic effects mediated by trogocytosis from the respective CAR’s antitumor activity by reducing CAR affinity (Fig. 1A). This assumption is supported by the recent finding that T cells expressing a CAR targeting CD19 (“CAT”) with ~ 40-fold lower affinity (*K*_on_ = 2.2 × 10^5 ^M^−1^ s^−1^, *K*_off_ = 3.1 × 10^−3^ s^−1^, *K*_D_ = 14 nM) than the clinically approved FMC63-based CAR (*K*_on_ = 2.1 × 10^5 ^M^−1^ s − 1, *K*_off_ = 6.8 × 10^−5^ s^−1^, *K*_D_ = 0.328 nM) exhibit higher efficacy and persistence than FMC63-based CAR T cells in a mouse model [9], as well as robust antitumor efficacy and persistence in two clinical trials [9, 10]. We hypothesize that reduced trogocytosis may have contributed to this enhanced in vivo persistence. We therefore generated non-binding (∆scFv), low affinity (CAT) and high affinity (FMC63) CD19 CAR T cells (Fig. 1B) in order to determine the impact of CAR affinity on trogocytosis. In addition, we explored the generalizability of this approach by using the CD229-specific CAR binding domain 2D3 and a single amino acid substitution variant with reduced affinity [11] (Fig. 1C) and determined the impact on trogocytosis (Fig. 1D). We found that low affinity CD19 and CD229 CAR T cells showed killing of target cells indistinguishable from high affinity receptors (Fig. 1E, F) as well as unchanged interferon-γ production and proliferation when stimulated by beads covalently coated with CD19 (Supplementary Fig. 1). Importantly, treatment with low affinity constructs was associated with significantly higher levels of the targeted antigen on the tumor cells at the end of in vitro co-cultures (Fig. 1G, H), suggesting reduced levels of antigen loss. We next explored whether this finding might be the result of reduced trogocytosis. Indeed, we found that high-affinity CD19 and CD229 CAR T cells had acquired membrane from their respective target cells during the co-culture, a hallmark of trogocytosis, but that use of low affinity CARs had significantly reduced this membrane transfer (Fig. 1I, J). Additionally, we found that use of low affinity CD19 (Supplementary Fig. 2A) and CD229 (Supplementary Fig. 2B) CARs had also resulted in substantially reduced transfer of the respective target antigen from the tumor cells to the CAR T cells. We further observed that, while target antigen levels were higher, CAR expression had decreased significantly on FMC63 but not CAT CAR T cells upon culture with lymphoma cells, possibly indicating increased CAR internalization of high affinity CD19 CAR T cells (Supplementary Fig. 3). Finally, in a short-term B cell acute lymphoblastic leukemia (B-ALL) mouse model (Fig. 1K) showing incomplete tumor control (Supplementary Fig. 4), we also observed significantly reduced CD19 expression on tumor cells from mice treated with FMC63 CAR T cells but not CAT CAR T cells. Taken together, these data show that it is possible to significantly limit trogocytosis by reducing CAR affinity while maintaining antitumor activity as well as clinical efficacy [9, 10].

**Fig. 1:**
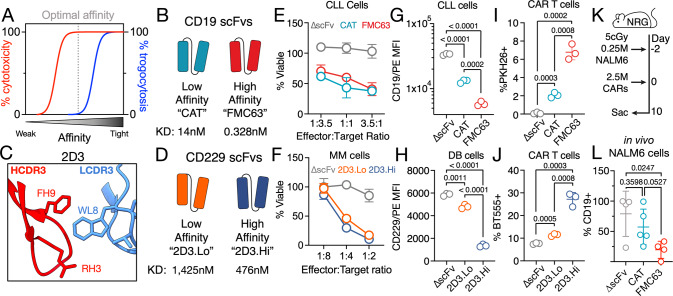
Low affinity CAR T cells exhibit reduced trogocytosis while preserving antitumor activity. **A** Schema of relationship between CAR affinity, cytotoxicity, and trogocytosis. **B** Schema of high and low affinity CD19 scFvs. **C** Structure of CDR3 regions and key residues of CD229 scFv 2D3 as determined by AlphaFold [11]. **D** Schema of high and low affinity CD229 scFvs. Low and high affinity CAR T cells were cultured with either **E** CD19+ primary CLL cells or **F** CD229+ multiple myeloma cell line U266 at the indicated effector:target ratios for 4 h. CLL cell viability was assessed by flow cytometry using counting beads. Multiple myeloma cell viability was determined by luminescence. **G** CD19 MFI on CLL cells, **H** CD229 MFI on DB cells, **I** % PKH26+ CAR T cells, and **J** % BioTracker 555 (BT555)+ CAR T cells following a 4 h coculture with CAR T cells and PKH26-labeled primary CLL cells or BT555-labeled DB cells at an effector:target ratio of 4:1. Data represent mean ± SD from three replicates. Statistical significance was determined by two-sided Student’s *t* test. **K** Schema of trogocytosis mouse model. **L** Percentage of CD19+ NALM6 tumor cells in the spleens of NRG mice treated with high and low affinity CD19 CAR T cells. Data represent mean ± SD from ∆scFv (*N* = 4) treated and FMC63 (*N* = 4) or CAT (*N* = 5) treated mice. Statistical significance was determined by two-sided Student’s *t* test.

We next explored whether limiting trogocytosis using low affinity CD19 CAR T cells would be an effective strategy across malignancies currently treated with high affinity CD19 CAR T cells. To this end we first determined the level of CAR T cell-mediated trogocytosis in five common B cell malignancies and found that all tumor cells experienced substantial levels of trogocytosis leading to the emergence of CD19^neg^ tumor cells when treated with high affinity CD19 CAR T cells (Fig. 2A). Determining CD19 expression in the aggressive lymphoma subtypes diffuse large B cell lymphoma (DLBCL, Fig. 2B) and mantle cell lymphoma (MCL, Fig. 2C) we again found that use of a low affinity CD19 CAR significantly reduced CD19 loss on the tumor cells (Fig. 2D, E), demonstrating that affinity modulation represents an effective strategy to reduce trogocytosis across B cell malignancies.

**Fig. 2:**
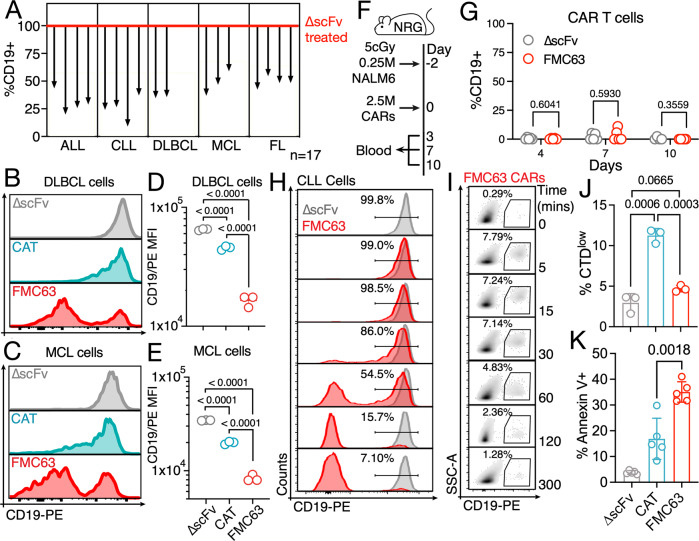
CAR-mediated trogocytosis is rapid and associated with targeting of CAR T cells. **A** CD19 expression on primary ALL, CLL, DLBCL, MCL, and FL cells following a 4-h coculture with FMC63 CAR T cells at an effector:target ratio of 4:1. Values are normalized to CD19 expression following coculture with ∆scFv CAR T cells. Example of CD19 staining and mean CD19 MFI on primary DLBCL (**B**, **C**) and MCL (**D**, **E**) samples following a 4 h coculture with CAR T cells at an effector:target ratio of 4:1. Data represent mean ± SD from three replicates. Statistical significance was determined by two-sided Student’s *t* test. **F** Schema of trogocytosis mouse model. **G** Percentage of CD19+ CAR T cells on days 4, 7, and 10 in the spleens of ∆scFv (gray, *N* = 5) or FMC63 (red, *N* = 5) CAR T cell treated NRG mice. Statistical significance was determined by two-sided Student’s *t* test. CD19 surface expression on **H** primary CLL cells and **I** FMC63 CAR T cells at different timepoints during co-culture as determined by flow cytometry. **J** Expansion of CellTrace dye (CTD) Far Red-labeled CD19 CAR T cells following a 24 h coculture with Raji cells at an effector:target ratio of 2.5:1 as determined by flow cytometry. Data represent mean ± SD from three replicates. Statistical significance was determined by two-sided Student’s *t* test. **K** Annexin V staining of ∆scFv (gray) or CD19 (red) CAR T cells following a 1.5-h coculture with Raji cells at an effector:target ratio of 1:1. Data represent mean ± SD from five replicates. Statistical significance was determined by two-sided Student’s *t* test.

Considering the substantial amount of antigen transferred to the CAR T cells observed in our in vitro experiments as well the previously described CAR T cell-mediated killing of T cells engineered to express CD19 [6], suggesting the possibility of fratricide, we next explored the effect of trogocytosis on the CAR T cells themselves. It has previously been shown in a mouse model as well as in a clinical trial that low affinity CD19 CAR T cells show superior persistence when compared to high affinity CAR T cells [9]. Multiple explanations for this observation are possible, such as more physiological receptor signaling preventing early exhaustion or activation-induced cell death and induction of a more stem-like T cell phenotype of low affinity CAR T cells [12, 13]. However, we hypothesize that increased persistence of low affinity CAR T cells may also be the result of reduced fratricide of CD19^pos^ CAR T cells due to lower levels of trogocytosis. In our aggressive B cell leukemia mouse model, we had observed that within 10 days of treatment with high-affinity CD19 CAR T cells, the majority of the remaining tumor cells had become CD19^neg^ suggesting downregulation or trogocytosis-mediated loss of CD19 in response to CD19 CAR T cell treatment (Fig. 1L, Supplementary Fig. 5). Exploring the possibility of trogocytosis in this model, we next analyzed CAR T cells from the same animals at multiple time points for the presence of CD19 (Fig. 2F). Surprisingly, we did not observe any CD19^pos^ CAR T cells in these animals throughout the experiment (Fig. 2G) either indicating that CD19 loss was not conferred by CAR-mediated trogocytosis or that CD19 CAR T cells had either lost the antigen or had undergone fratricide. To explore the latter possibilities, we next determined how long CD19 remains detectable on the surface of CD19 CAR T cells following trogocytosis by performing a time-course analysis of high-affinity CAR-mediated trogocytosis. We found that trogocytosis occurs rapidly as an almost complete loss of CD19 from tumor cells was observed within 2 h (Fig. 2H). Importantly, while loss of CD19 on tumor cells increased over time, a population of CD19^pos^ CAR T cells became detectable within 5 min but immediately started to decline (Fig. 2I), indicating that, following trogocytosis, CD19 was indeed either lost, for example via shedding or internalization, or CD19^pos^ CAR T cells had undergone fratricide. While we cannot rule out that CAR T cells internalize or shed acquired CD19 antigen, we found that, at the end of a 24 h co-culture with CD19^pos^ tumor cells, expansion of high affinity CD19 CAR T cells was significantly reduced compared to low affinity CAR T cells (Fig. 2J, Supplementary Fig. 6), although both had proliferated at comparable rates when stimulated with CD19-coated beads (Supplementary Fig. 1B), and that high affinity CAR T cells showed increased staining with the apoptosis marker Annexin V compared to low affinity CAR T cells (Fig. 2K), indicating increased targeting by bystander CD19 CAR T cells.

Taken together, we show that high affinity CD19 CAR T cells confer substantial antigen loss in many of the major tumor types currently approved or under clinical investigation for treatment with high affinity CD19 CAR T cells, potentially fueling a reservoir of persistent antigen-negative tumor cells. We further show that is possible to robustly separate trogocytosis and its deleterious effects from antitumor activity by using low affinity CAR constructs. In addition to reduced trogocytosis, there may be other benefits to using low-affinity CAR binding domains, such as increased selectivity [14] and reduced exhaustion [15]. We hypothesize that while the identification of low affinity constructs with equal antitumor activity may not be possible in all settings, e.g., due to low antigen density on the respective tumor cells, trogocytosis represents a key parameter that should be assessed carefully in all novel CAR constructs.

## Supplementary information

Supplementary Material
