# Supplementary material for: Low-affinity CAR T cells exhibit reduced trogocytosis, preventing rapid antigen loss, and increasing CAR T cell expansion
Source: Leukemia. 2022 Apr 30;36(7):1943–6. doi: 10.1038/s41375-022-01585-2 (PMC9252916; doi:10.1038/s41375-022-01585-2)
Supplement: Supplementary file 1 — Supplementary Material [file 41375_2022_1585_MOESM1_ESM.docx]

**SUPPLEMENTARY FIGURES**


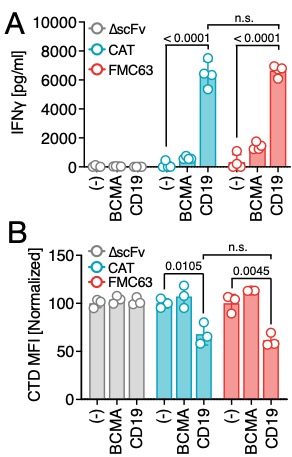


**Supplementary Figure 1: Comparable cytokine production and proliferation of low and high affinity CD19 CAR T cells. (A)** CAR T cells were thawed and cultured in the presence of 40IU/mL IL2 before addition of CD19-coupled magnetic beads. After 48 hours, supernatants were harvested and IFNγ concentrations determined by ELISA. Data represent mean ± SD from four replicates. **(B)** CAR T cells were cultured for 5 days without IL2 before staining with CellTrace Far Red dye and addition of CD19-coupled magnetic beads and 40IU/mL IL2. Dye dilution was determined after 5 days by flow cytometry. Data represent mean ± SD from three replicates. Statistical significance was determined by two-sided Student’s *t* test.

**Supplementary Figure 2: Use of low affinity CAR T cells reduces the amount of target antigen transferred to CAR T cells. (A)** % CD19+ CAR T cells following a 15 minute coculture with primary CLL cells at an effector:target ratio of 1:10. **(B)** Presence of CD229 on CAR T cells alone or when co-cultured with CD229+ U266 cells for 1h at an effector:target ratio of 1:2. Data represent mean ± SD from three replicates. Statistical significance was determined by two-sided Student’s *t* test.

**Supplementary Figure 3: CAR surface expression is significantly decreased in high affinity CAR T cells upon coculture with lymphoma cells.** CAR surface expression on CAT and FMC63 CAR T cells following a 4-hour coculture with primary CLL cells at an effector:target ratio of 1:1. CAR expression was assessed by staining for an N-terminal HA tag and data were normalized to CAR expression on untreated CAR T cells. Statistical significance was determined by two-sided Student’s *t* test.

**Supplementary Figure 4:** **Incomplete control of NALM6 tumor in mice treated with a low dose of high affinity CD19 CAR T cells.** IVIS signal in NALM6-luc bearing mice treated with ∆scFv (top) or CD19 (bottom) CAR T cells following D-luciferin injection.

**Supplementary Figure 5: CD19 surface expression is reduced on NALM6 cells from mice treated with high-affinity CD19 CAR T cells.** CD19 mean fluorescence intensity (MFI) on total (left) or CD19^pos^ (right) NALM6 tumor cells in the spleens of NRG mice treated with high and low affinity CD19 CAR T cells. Data represent mean ± SD from ∆scFv (N=4) treated and FMC63 or CAT (N=5) treated mice. Statistical significance was determined by two-sided Student’s *t* test.

**Supplementary Figure 6: Dye dilution of CD19 CAR T cells during co-culture with CD19^pos^ B cell lymphoma cells.** Primary human low and high affinity CD19 CAR T cells and T cells expressing a CAR without a binding domain were labelled with CellTrace Far Red dye (CTD, Thermo) and then cocultured with CD19^pos^ Raji cells for 24h. Cocultures were stained for CD3 and CTD^low^ percentages determined by flow cytometry. Data are representative plots of individual technical replicates shown in Fig. 2J.

**Supplementary Figure 7: Surface expression and transduction efficiency of CD19 CAR constructs.** Primary human lymphocytes were transduced using ∆scFv, CAT, and FMC63-based CAR constructs and analyzed for surface expression by anti-HA staining using flow cytometry after manufacturing. Average mean fluorescence intensity (MFI) values  ± SD were calculated from three replicates.


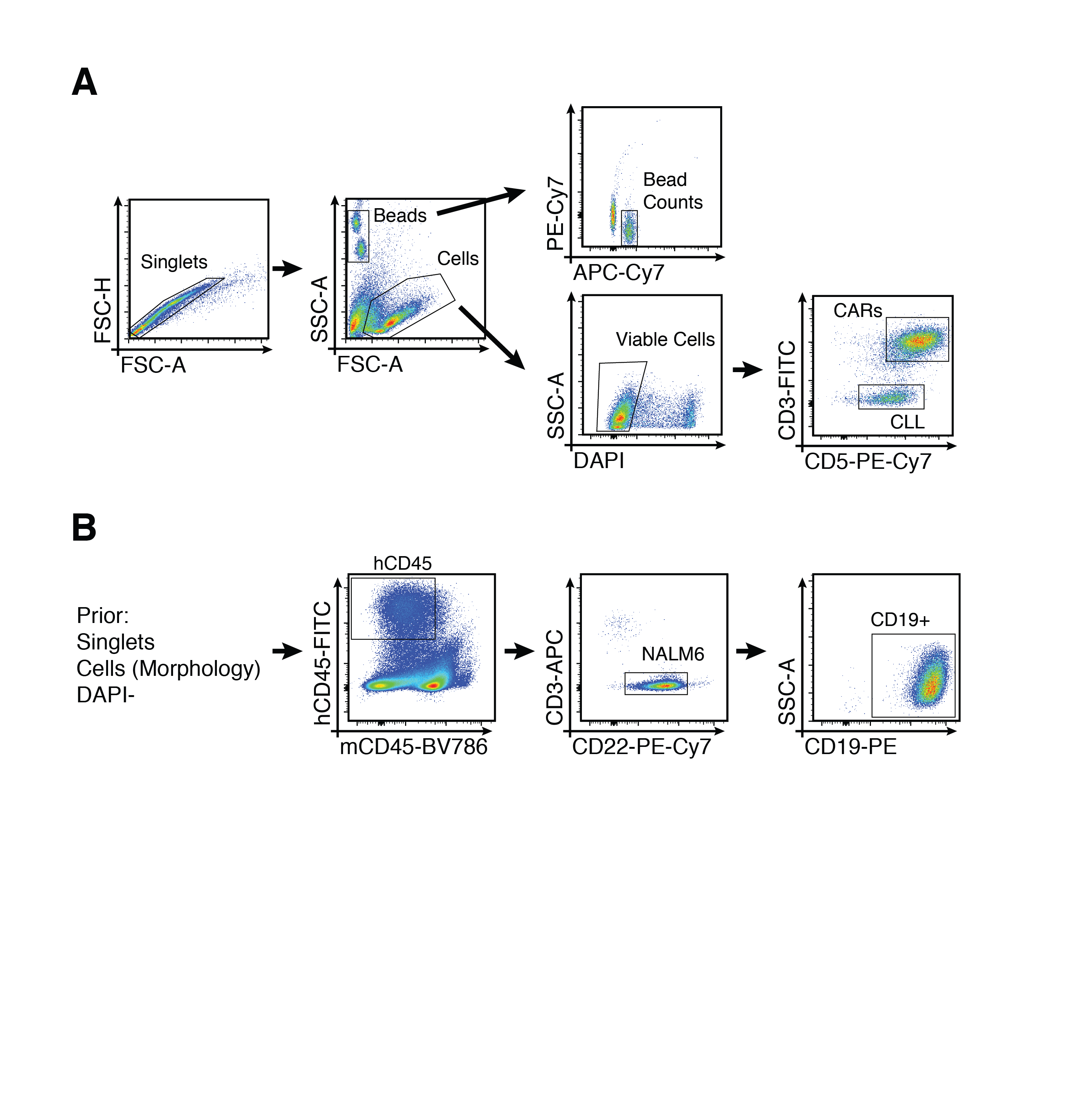


**Supplementary Figure 8: Flow cytometry gating schemes. (A)** Gating scheme for *in vitro* cytotoxicity and trogocytosis assays. **(B)** Gating scheme for *in vivo* trogocytosis assay, cells are pre-gated on singlets, cell morphology and DAPI- as shown in A.

**SUPPLEMENTARY TABLES**

**Supplementary Table 1: Antibodies used for flow cytometry**

**SUPPLEMENTARY METHODS**

**Cell lines and primary samples**

NALM6 cells were purchased from ATCC and cultured at 37˚C/5% CO_2_ in ATCC-formulated RPMI-1640 (Gibco #A10491-01) supplemented with 10% FCS (Gibco #10437028) and 50U/mL penicillin/streptomycin. NALM6 cells were transduced with pHIV-Luc-ZsGreen lentivirus (Addgene #39196) and sorted by FACS prior to use. All cell lines were authenticated by ATCC. NALM6 cells used in in vivo experiments were authenticated by short tandem repeat profiling. Cell lines used in this study were confirmed to be mycoplasma negative by PCR. No commonly misidentified cell lines were used in this study. Primary patient samples were prepared as follows: whole blood or bone marrow aspirate was collected and mononuclear cells were isolated by density gradient using Ficoll-Paque (GE #17-1440-02), samples were cryopreserved until analysis.

**CAR constructs and virus production**

CAR T cells were generated using lentiviral or gammaretroviral transduction. Second generation CAR constructs based on FMC63 ^1^, CAT ^2,3^, or 2D3 ^4^ using the 4-1BB costimulatory and CD3ζ signaling domains, as well as the CD8α hinge and transmembrane domains were cloned into pRRLSIN.cPPT.PGK-GFP.WPRE (Addgene # 12252) or SFG.CNb30_opt.IRES.eGFP (Addgene # 22493) backbones. VSV-G-pseudotyped lentivirus was obtained by co-transfection of Lenti-X 293T cells (Takara) with pRRL-based transfer plasmids as well as pMD2.G (Adgene # 12259) and psPAX2 (Addgene # 12260) using Lipofectamine 2000 (Thermo) according to the manufacturer’s instructions. Amphotropic gammaretrovirus was generated by transfection of Phoenix-Ampho cells (ATCC # CRL-3213) with SFG-based transfer plasmids using Lipofectamine 2000 according to the manufacturer’s instructions. Virus-containing supernatants were concentrated with Lenti-X Concentrator or Retro-X concentrator (Takara), respectively, and 24 well plates coated with Retronectin (Takara # T100B) were incubated with concentrated viruses according to the manufacturer’s instructions.

**CAR T cell production**

Buffy coats from healthy donors were obtained from the Blood Centers of America or the New York Blood Center and peripheral blood mononuclear cells (PBMC) were isolated by density gradient using Ficoll-Paque. T cells were cultured in AIM V (Thermo) supplemented with 5% human serum (Sigma #H3667). PBMCs were stimulated for 2 days with CD3/CD28 T cell activation beads (Thermo # 11131D) in the presence of 40IU/mL IL2 (R&D Systems # 202-IL-010) and incubated at 37°C/5% CO_2_. Bead-stimulated cells were transferred to Retronectin-coated virus-containing plates and incubated overnight. Transduction was repeated the next day before counting and diluting cells to 0.4x10^6^ cells/ml. After the second transduction cells were grown for an additional 7 days before removing beads using a DynaMag-15 magnet (Thermo). IL-2 was replenished every 2 days to 40IU/mL. Cells were frozen in 90% FCS/10% DMSO in liquid nitrogen until needed. CAT and FMC63-based CAR T cells showed comparable surface expression levels on primary human T cells after manufacturing (Suppl. Fig. 7). If transduction rate between constructs differed by more than 5%, all CAR T cell products were FACS sorted to achieve 95% CAR+ populations.

***In vitro* trogocytosis assay**

CAR T cells were cocultured with target cells at a defined effector:target ratio. For some experiments, target cells were first labelled with membrane dyes PKH26 (Sigma # MINI26) or BioTracker 555 (Sigma # SCT107) according to the manufacturer’s instructions. Cells were stained with the antibody panel described in Supplemental Table 1, 500ng/mL DAPI (Invitrogen # D1306) and counting beads (Thermo # PCB 100) were added following staining and cells were analyzed on a LSRFortessa or an LSR II flow cytometer (BD). Effector:target ratios and coculture times are listed in the figure legends. Gating schemes can be found in Suppl. Fig. 8.

***In vivo* trogocytosis model**

7-8-week-old female NSG mice were randomly grouped and sublethally irradiated at 5cGy on the morning of day -2 followed by i.v. administration of 2.5x10^5^ NALM6-Luc cells. Mice were administered 3.3 mg D-Luciferin in the morning of day 3 and luminescence was recorded after 10 minutes by an In Vivo Imaging System (IVIS, Perkin Elmer) followed by i.v. injection of 2.5x10^6^ ∆scFv, CAT, or FMC63 CAR T cells. Peripheral blood samples were obtained from all animals on days 3, 7, and 10. All injections and monitoring by IVIS were performed by the preclinical research resource core at the University of Utah, a blinded third party. Tumor progression was monitored by IVIS on the morning of day 10 and spleens and cervical lymph nodes were collected in the afternoon. Lymph nodes were stained for CD19 expression using the panel described in supplemental table 1 following incubation with FcR blocking reagent (Miltenyi Biotec # 130-092-575). Cells were analyzed on a BD LSRFortessa. Gating schemes can be found in Suppl. Fig. 8.

**CD19 bead stimulation assays**

For CAR T cell proliferation assays, ∆scFv, FMC63, and CAT CAR T cells were stained with CellTrace Far Red dye (Thermo # C34564) according to the manufacturer’s instructions. CAR T cells were starved of IL-2 for 5 days before the addition of CD19-coated (Acro Biosystems # MBS-K005) or BCMA-coated (Acro Biosystems # MBS-K004) paramagnetic beads. 40IU/mL IL-2 was added to cultures together with beads, and replenished every 2 days. Dye dilution was determined after 5 days on an LSR II flow cytometer (BD). IFNγ concentrations in cell culture supernatants were determined after 48h by ELISA (Biolegend # 430104) according to the manufacturer’s instructions. Flow cytometry data was analyzed using FlowJo 10 (BD).

**Luciferase-based cytotoxicity assay**

To determine the cytotoxicity of variant CD229 CAR T cells against the multiple myeloma cell line U-266, U-266 cells were transduced with pHIV-Luc-ZsGreen lentivirus and sorted on a FACSaria flow cytometer (BD) for GFP expression. 5×104 target U-266 cells were seeded in each well of a round bottom 96 well plate. Various ratios of CAR T cells were co-cultured with the U-266 cells overnight at 37°C 5% CO2. After the co culture, cells were suspended by gentle pipetting and 100uL were moved to a 96 well black flat bottom plate. 150 μg/ml D-luciferin (Gold Biotechnology Cat# LUCNA-2G) was added to the cells and incubated for 5 mins at 37°C. Luminescence was determined on a multi-mode plate reader (Tecan Spark).

**Study approval**

Informed consent was obtained from all patients and samples were collected with the approval of the Institutional Review Boards of the University of Utah (IRB #45880) and Tisch Cancer Institute (IRB #13-1347, 17-2164, 17-0554). All animal procedures were conducted under protocol #16-05007, approved by the Institutional Animal Care and Use Committee at the University of Utah.

**Statistical analyses**

No statistical methods were used to determine sample size. Sample sizes for in vivo experiments were based on previous experiments and similar published studies. No data were excluded from analysis and all attempts at replication were successful. Animals were age- and sex-matched and randomly grouped on the day of xenograft administration. CAR T cell administration, IVIS measurements and tissue collection were performed by a blinded third party, namely the Preclinical Research Resource core at the Huntsman Cancer Institute. Significance of differences in cell numbers, cytokine levels, and mean fluorescence intensity were calculated by two-tailed Student’s t-test. All statistical tests were performed using Prism 9 (GraphPad Software). Results were considered significant when p or adjusted p < 0.05.

**REFERENCES**

1 Nicholson, I. C. *et al.* Construction and characterisation of a functional CD19 specific single chain Fv fragment for immunotherapy of B lineage leukaemia and lymphoma. *Mol Immunol* **34**, 1157-1165 (1997).

2 Ghorashian, S. *et al.* Enhanced CAR T cell expansion and prolonged persistence in pediatric patients with ALL treated with a low-affinity CD19 CAR. *Nature Medicine* **25**, 1408-1414, doi:10.1038/s41591-019-0549-5 (2019).

3 AMROLIA P, C. G., GHORASHIAN S, Kramer A, MEKKAOUI L, Pule M. Chimeric antigen receptor (CAR) comprising a CD19-binding domain. US patent (2019).

4 Radhakrishnan, S. V. *et al.* CD229 CAR T cells eliminate multiple myeloma and tumor propagating cells without fratricide. *Nat Commun* **11**, 798, doi:10.1038/s41467-020-14619-z (2020).
